# Brown-Séquard: On neural networks and brain localization of functions

**DOI:** 10.1590/S1980-57642014DN81000012

**Published:** 2014

**Authors:** Eliasz Engelhardt

**Affiliations:** 1 Neurologist, Full Professor (retired), Cognitive and Behavioral Neurology Unit, Institute of Neurology Deolindo Couto – Institute of Psychiatry – Center for Alzheimer Disease (CDA-IPUB), Federal University of Rio de Janeiro (UFRJ), Rio de Janeiro RJ, Brazil.

**Keywords:** neural networks, network of anastomosing cells, dynamogenesis, excitatory activity, inhibitory activity, action at a distance, recovery

## Abstract

The notion that the brain (encephalon) is a network of interconnected neurons has a long and memorable history. Cytoarchitectonic and hodological studies coupled with advanced neuroimaging techniques have produced a substantial body of knowledge on structural and functional organization. Acquiring the rich knowledge held today took a long and winding journey. Important advancements were made in the 19^th^ century, with the remarkable Brown-Séquard figuring as one of the protagonists. Regarding the brain, he proposed nine mental and physical functions (organs) related to distributed cell clusters, interconnected according to their roles, the "network of anastomosing cells", dynamically submitted to "dynamogenic and inhibitory activities", and "action at a distance" concepts, the latter also related to his notion of "recovery". It is remarkable that someone was able to propose, ahead of his time, and with the limited technical resources available, such significant concepts that paved the way for the current state of knowledge.

## Abstract (pt)

A noção que o cérebro (encéfalo) é uma rede de neurônios interconectados possui uma história longa e memorável. Estudos citoarquitetônicos e hodológicos acoplados a técnicas avançadas de neuroimagem produziram uma quantidade expressiva de material sobre sua organização estrutural e funcional. Chegar ao rico conhecimento atual requereu percorrer um longo e penoso caminho. Passos importantes começaram no século 19, tendo o notável Brown-Séquard como um dos protagonistas. Considerando o cérebro ele propôs nove funções (órgãos) mentais e físicos relacionados a conglomerados celulares distribuídos, interconectados de acordo com suas ações, a "rede de células anastomosadas", submetida dinamicamente aos conceitos da "atividades dinamogênicas e inibidoras" e "ação à distância", este também relacionado à sua noção de "recuperação". É incrível uma pessoa ter sido capaz de propor, adiante do seu tempo, com os limitados recursos técnicos disponíveis, conceitos tão significantes que prepararam o caminho para o conhecimento atual.

## INTRODUCTION

Information in the brain, is viewed among present-day neurosciences as being represented and processed by distributed groups of functionally interconnected neurons - a neural network.^1,2^ Thus, a brain network comprises a set of neural elements, portions of cortical and subcortical gray matter regions, connected together by structural and functional networks that can be revealed by diffusion tensor and functional magnetic resonance imaging.^2-4^

Achieving the rich knowledge held today, now acquired using sophisticated techniques, was a long and arduous journey. The first steps were taken in the 19^th^ century with Brown-Séquard figuring as one of the most prominent protagonists.

## THE PERSONAGE

Charles-Édouard Brown-Séquard was born in Mauritius and ended his life in Paris (1817-1894). He (and his mother) left for France (1838), where he completed the baccalaureate and acquired his doctoral degree at the *Faculté de Médecine* (Paris) (1846), presenting a thesis on the spinal cord *(Recherches et expériences sur la physiologie de la moelle épinière)*. He changed his baptismal name (Charles Édouard Brown) by adding his mother's maiden name to his own after her death, in 1842.5-8 He was a physiologist, practiced clinical medicine, mainly in the neurological field, and was a communicative lecturer.^8-10^ He maintained close relationships with several renowned personalities of the day, including Broca, Charcot, Darwin, Hall, Huxley, Jackson, von Monakow, Pasteur, Sherrington, Vulpian, among others, with whom he corresponded, discussed scientific issues, and exerted influence.^6,11^

## THE RESEARCHER AND HIS FINDINGS

Brown-Séquard was a keen observer and experimentalist, applying vivisection in his research. His studies included neurological examination of patients, and eventually pathological observations. He contributed greatly to knowledge on the central nervous system, as well as its autonomic division, and to sense organs. Clinical disorders of the spinal cord (syndrome of its hemi-section [1850]) and of the brain (aphasia and epilepsy), were also his areas of expertise. Endocrinology merits special attention, besides other varied topics.^10-12^

## THE BRAIN: NETWORKS, NOT CENTERS

At the time, many physiologists and clinicians advocated that most of the functions of the brain were related to centers (or organs), distinct and well-defined cell clusters to which the functions were confined, constituting the "theory of centers". This localizationist stance, based on experimental and/or clinical observations, was shared by famous personalities such as Fritsch, Hitzig, Ferrier, Luciani, Charcot, among many others.^6,13,14^ Brown-Séquard, based on well-founded experimental and/or clinical research, strongly rejected this view, not believing in brain localization, as it was accepted at the time, and fighting against these doctrines and their supporters.^15^ He was not alone in this anti-localizationist battle, having the company of influential personalities such as Goltz, Jackson, von Monakow, Sherrington, and others who shared similar opinions.^6,13,14^

Brown-Séquard's experimental work led him to propose the concept of a *réseau de cellules anastomosées* ("network of anastomosing cells"), occupying the entire surface of the hemispheres, in substitution of the "theory of centers", explaining: "Nerve cells endowed with any of the cerebral functions, instead of forming clusters, as is supposed, are disseminated through the whole encephalon [in such a way that they are everywhere associated to one another by fibers that permit them to act together], so that no local lesion or irritation can reach more than a part of those endowed with the same function or the same kind of activity".^15-19^

## THE NETWORKS AND THEIR FUNCTIONAL BASIS

The functional anatomy of the brain (encephalon) was envisioned by him as an aggregate of nine distinct disseminated organs richly and dynamically interconnected, and that each organ was organized, not as topographically isolated clusters of neurons, but as widely disseminated networks. The activity of these organs was related to nine mental and physical functions.^15,18^ (Box 1). These functions include mental faculties, especially the intellect (located chiefly in the convolutions), memory, faculties of expressing ideas, as well as perceptions and motor functions. Some disorders such as aphasia, word blindness and deafness, agraphia, and loss of consciousness (attributed to inhibition) were also considered.^15,17,18^

**Box 1:** Brain functions, mental and physical, according to Brown-Séquard.^18^

(i)	intelligence
(ii)	consciousness
(iii)	the faculties of expressing ideas by speech, writing, and gesture
(iv)	memory
(v)	vision, audition, olfaction, taste, touch, and the common kinds of general sensitivity
(vi)	muscular sense
(vii)	voluntary movements
(viii)	respiratory movements
(ix)	deglutition

Brown-Séquard's "networks" are submitted to *activités dynamogeniques et inhibitrices* ("dynamogenic [excitatory] and inhibitory activities"), as follows : "Decreases or increases in activity generally or even always coexist, and the same excitation of a point of the nervous system which propagates at a distance, and produces inhibition of one activity in certain parts of the nervous system, produces also dynamogenesis (excitation) in other parts".^13,15,17-20^

The statement above already embraces the concept of *action à distance* ("action at a distance") which he included in the investigation of cerebral localization: "I do not believe that the lesion itself has any direct bearing. On the contrary, I contend that the observed clinical phenomena in the various brain diseases are due to irritation or inhibition effects provoked in distant areas of the brain by the tissue surrounding the lesion site".^8,15,17,20^ He pointed to three different mechanisms behind symptoms secondary to local alterations:

"(i) direct results of injury or disease, producing loss of function or irritation;
(ii) alterations in the quantity or quality of the blood, and
(iii) reflex influence, the starting point of which may be in an altered part of the brain or in any part of the sensitive or centripetal nerve".^21^

Brown-Séquard explained how the disease in one part of the brain could disrupt the function of distant parts: "We currently know that disease in the hemispheres of the brain may be followed by alterations of nutrition in the pons Varolii, medulla oblongata, spinal cord, among other structures...we can easily conceive that a disease of any part of the brain could lead to alterations in circulation and nutrition in other parts of this organ itself, and thereby a loss of this or that function".^15,18^ He attributed the observed dysfunctions to "some peculiar influence exerted at a distance from some parts of the cerebral lobes on the active lower parts of the brain"(Box 2).^20,21^

**Box 2:** Brown-Séquard's basic concepts underlying the brain structure and function.^15,17-20^

**Organs** *(organes) *- basic elements of the functional anatomy of the encephalon, constituted by aggregates of nine distinct disseminated neuron clusters (organs) dynamically interconnected.
**Network of anastomosing cells** *(réseau de cellules anastomosées) *- nerve cells possessing the same functions communicate with each other, such communications being essential, and that concerted and harmonious actions can take place by means of intervening fibers both between distant and neighboring nerve cells.
**Functions** *(fonctions)* - the activity of these dynamically interconnected organs exert nine mental and physical functions (Box 1).
**Action at a distance** *(action à distance)* - a lesion in one part of an organ temporarily inhibits elements distant from the organ, by “some peculiar influence exerted at a distance”.
**Inhibitory activities** *(activités inhibitrices)* - decrease or arresting of activity that may be exerted by certain organs on others, put in action in a direct or reflex manner; and can depress all activities and properties (normal and morbid).
**Dynamogenic (excitatory) activities** *(activités dynamogeniques) *- increase or intensification of activity that may be exerted by certain organs on others, put in action in a direct or reflex manner, and can enhance the energy of all activities and properties (normal and morbid).
**Relationships between inhibition and dynamogenesis **- decrease or increase in activity; the same excitation of a point of the nervous system which propagate at a distance, and produces inhibition of one activity in certain parts of the nervous system, may also produce excitation in other parts.
**Recovery** *(récupération) *- remaining tissue after a lesion, and/or the release of the inhibition of undamaged distant structures, may result in functional recovery.

Brown-Séquard dedicated special attention to "recovery" of brain functions based on the "action at a distance" concept: "A lesion of one part of an organ temporarily inhibits elements distant from the organ, and that the release of the inhibition of these undamaged distant elements may result in recovery." Adding: "There is no doubt that any destruction of brain tissue, however small, takes away contributors to several functions. But the nervous elements remaining are evidently sufficient for the performance of these functions." Thus, he agreed with the clinical observation that focal lesion may produce loss of function, but that recovery means that a certain amount of undamaged tissue is capable of assuming the lost function.^14,15,17,18^

The "dynamogenic and inhibitory activities" and the "action at a distance" concepts permitted Brown-Séquard, based on clinical-pathological cases, to put forward three propositions to summarize these activities, providing analogies with peripheral nerve actions, and example clinical conditions (Box 3).^22^

**Box 3:** Brown-Séquard's three propositions of organized brain function according to clinical-pathological based studies.^22^

1^st^. All symptoms of organic disorders of the encephalon, when the lesion involves any part beyond the cells from where fibers arise…, even when they constitute two distinct groups [characterized, one by the cessation of an activity*, the other by the manifestation of an activity**], are the effects of an influence exerted on more or less distant parts by an irritation which had its origin at the seat of the lesion or in its vicinity;
2^nd^. The mechanism of production of symptoms characterized essentially by a *cessation of activity *(such as *paralysis, anesthesia, amaurosis, aphasia, loss of consciousness, *etc.) is identical to the stopping of the heart by irritation of the vagus nerve, and consists of an irritation beginning at the injured point of the encephalon, propagating from there to the cells whose function will disappear and produce a more or less complete arrest of their activity at the site;
3^rd^. The mechanism of production of phenomena which consist of a *manifestation of activity *(such as *delusions, epileptiform convulsions, chorea or other contractions, tremors, vomiting, hiccups, *etc.) is the same by which all these phenomena are produced, as their primary cause is a peripheral irritation, either from the skin or mucous membranes, or of a part of a centripetal nerve.

* inhibitory activity;

** dynamogenic or excitatory activity

## COMMENTS

Brown-Séquard was an early protagonist who proposed that the brain is constituted by functional and structural networks. Despite the use of a wide variety of different experimental animals, irrespective of their evolutionary level, and consequent distinctive characteristics, his powers of insight should be recognized, as well as his sound theoretical inferences on brain function.^6,10,13,15,17,18,23^ He proposed nine mental and physical functions (organs) related to distributed cell clusters interconnected according to their properties (endeavors), underpinned by the "network of anastomosing cells"^10,15,17,18^ allied with the "dynamogenic and inhibitory activities" and "action at a distance" concepts,^15,17-19^ which provided an explanation for how disease in one part of the brain could turn off the function of distant parts, with consequent loss of varied functions.^14,18,21^ For functional "recovery" , he assumed the possibility that it was supported by the presence of remaining tissue after a lesion, and/or by the release of inhibition of undamaged distant elements.^15,17^

These assertions show resemblances with von Monakow's theories, including the "diaschisis" concept,^24^ and of Jackson's thoughts (1882), which implied an anatomical and physiological hierarchy of higher and lower centers, with the higher ones suppressing the function of the lower ones,^10,25^ if not a precursor announcement.

Later, following the advent of advanced neuroimaging techniques, the neural network concept gained ground and a deeper understanding about its structure and function^2,3^ emerged permitting the proposed existence of direct neuroanatomical connections between brain regions to facilitate the ongoing interregional neuronal communication.^2,4,26^ In the same way, nine consistently found "functional communities" were retrieved, related to well-known anatomical white matter tracts that interconnected most of these, reflecting the underlying structural connectivity architecture of the human brain.^26^

Is there a possibility that these nine "functional communities" reflect the same nine of Brown-Séquard's brain functions?

The following statement seems to be the best and most consolidated way to define this complex subject with present-day knowledge: "Our brain is a network, and consists of spatially distributed, but functionally linked regions that continuously share information with each other."^27^

An apparent resemblance, to some extent, can be seen in what Brown-Séquard envisioned.

In conclusion, it must be said with admiration how remarkable it is that a researcher with such limited technical resources was able to propose, ahead of his time, such significant concepts that paved the way for the present state of knowledge on the role of neural networks and brain function.
